# Lactobacillus rhamnosus HN001 Ameliorates BEZ235-Induced Intestinal Dysbiosis and Prolongs Cardiac Transplant Survival

**DOI:** 10.1128/spectrum.00794-22

**Published:** 2022-07-11

**Authors:** Xiaolong Miao, Yuancong Jiang, Deqiang Kong, Zelai Wu, Han Liu, Xiaolin Ye, Weihua Gong

**Affiliations:** a Department of Surgery, Second Affiliated Hospital of School of Medicine, Zhejiang University, Hangzhou, China; b Städtisches Klinikum Wolfenbüttel, Wolfenbüttel, Federal Republic of Germany; c Liangzhu Laboratory, Zhejiang University Medical Center, Hangzhou, China; Huazhong University of Science and Technology

**Keywords:** allograft rejection, BEZ235, intestinal microbiota dysbiosis, probiotic supplementation, propionic acid

## Abstract

Cardiac allograft rejection remains a major factor limiting long-term engraftment after transplantation. A novel phosphoinositide 3-kinase (PI3K)/mTOR dual inhibitor, BEZ235, prolonged cardiac allograft survival by effectively suppressing activation of the PI3K/serine/threonine kinase (AKT)/mTOR pathway. However, long-term usage of pharmacological immunosuppressant drugs can cause intestinal microbiota dysbiosis. We established mouse models of allogeneic heterotopic heart transplantation with different treatments. Fecal samples were collected and subjected to 16S rRNA sequencing and targeted fecal metabolomic analysis. Graft samples were taken for immune cell detection by flow cytometry. Inflammatory cytokines in serum were quantified by enzyme-linked immunosorbent assay (ELISA). Compared to single-target approaches (IC-87114 and rapamycin), BEZ235 more efficiently prolongs cardiac transplant survival. Interestingly, BEZ235 reduces the diversity and abundance of the intestinal microbiota community. We demonstrated that Lactobacillus rhamnosus HN001 rescues the intestinal microbiota imbalance induced by BEZ235.

**IMPORTANCE** Our data confirmed that the combination of BEZ235 and Lactobacillus rhamnosus HN001 significantly prolongs cardiac transplant survival. A main metabolic product of Lactobacillus rhamnosus HN001, propionic acid (PA), enriches regulatory T (Treg) cells and serves as a potent immunomodulatory supplement to BEZ235. Our study provides a novel and efficient therapeutic strategy for transplant recipients.

## INTRODUCTION

Acute rejection is the most important factor for long-term graft outcome, and it represents a significant therapeutic challenge (1). Small-molecule inhibitors of p110δ and p110α, such as IC-87114, prevent acute heart allograft rejection in a murine heart transplantation model by reducing proinflammatory factor expression (2). Rapamycin, a mammalian target of rapamycin (mTOR) inhibitor, has been used in acute heart allograft rejection (3). Rapamycin binds first to FKBP12, a prolyl isomerase, and then the rapamycin-FKBP12 complex binds to mTOR. The class IA phosphoinositide 3-kinase (PI3K) isoform p110δ is preferentially expressed in leukocytes and is crucial for CD4-positive (CD4^+^) T-cell growth and differentiation (4, 5). IC87114 is a selective inhibitor of PI3Kδ. Although single-target approaches (IC-87114 and rapamycin) have been developed to treat allograft rejection, their efficacy is not very satisfactory. Therefore, it is urgent to identify novel and effective treatments for preventing acute heart allograft rejection.

BEZ235, an imidazo(4,5-*c*) quinoline derivative, inhibits PI3K (p110-α, -β, -γ, and -δ isoforms) and mTOR kinase activity by binding to the ATP-binding cleft of these enzymes (6). BEZ235, with its dual PI3K-mTOR inhibitor activity, has been shown to have a significant antitumor effect on many cancers (7, 8). BEZ235 has been found to inhibit the proliferation of multiple myeloma and contribute to apoptosis by inducing autophagy (9). In addition, BEZ235 showed its potential use in graft-versus-host disease prophylaxis by improving the survival of the allogeneic hematopoietic stem cell transplant (10). BEZ235 is therefore a promising antirejection agent. Our previous study showed that, compared to single-target approaches (IC-87114 and rapamycin), BEZ235 efficiently prolongs cardiac transplant survival. However, BEZ235 also has adverse effects. It has been suggested that BEZ235 exerts detrimental effects via metabolic disturbances through mTOR pathway inhibition (11). At present, the mechanisms of BEZ235-induced metabolic disorders remain unclear.

The intestinal microbiota (IM) is involved in many physiological processes, such as nutrient absorption and substrate metabolism (12–14). Moreover, the IM plays a critical role in shaping systemic immune responses (15–17). The IM composition affects host immunity, so it is important to have a balanced IM. Probiotics have shown beneficial effects on the host immune response by manipulating the intestinal ecosystem (18, 19). Lactobacillus rhamnosus HN001 is a safe probiotic strain that has anti-inflammatory effects and modulates host immunity, thus showing health-promoting efficacy (20–22). The strain has specific functions related to gut barrier integrity (23), microbial community structure (24), and host metabolism (25). Supplementation with Lactobacillus rhamnosus HN001 has been found to significantly restore IM balance and reduce intestinal inflammation (26). It would be interesting to know whether a microbiota-targeted therapy could ameliorate metabolic disturbances induced by mTOR pathway inhibition. Nevertheless, the underlying mechanism of microbiota-targeted therapy is insufficiently understood.

Short-chain fatty acids (SCFAs), primarily acetate, propionate, and butyrate, are products of the microbial fermentation of dietary fibers. Several empirical studies have suggested that SCFAs have potential beneficial effects on host metabolism and cardiac health (27, 28). Propionic acid (PA) has been shown to modulate the disease course in autoimmune diseases by modulating the functions of regulatory T (Treg) cells (29, 30). This finding raises the hypothesis that a microbiota-targeted therapy may work by modulating metabolite production by the microbiota.

In this study, we observed that supplementation with Lactobacillus rhamnosus HN001 could rescue the IM imbalance induced by BEZ235 and reduce serum interleukin-6 (IL-6) levels in transplant recipient mice. Moreover, the combination of BEZ235 and Lactobacillus rhamnosus HN001 significantly prolonged cardiac transplant survival.

A main metabolic product of Lactobacillus rhamnosus HN001, PA, can enrich Treg cells and serve as a potent immunomodulatory supplement to BEZ235. These results demonstrated that there is a close relationship between cardiac allograft rejection, BEZ235-induced metabolic disorders, and gut microbial metabolism.

## RESULTS

### Impact of BEZ235 treatment on allograft survival.

**FIG 1:**
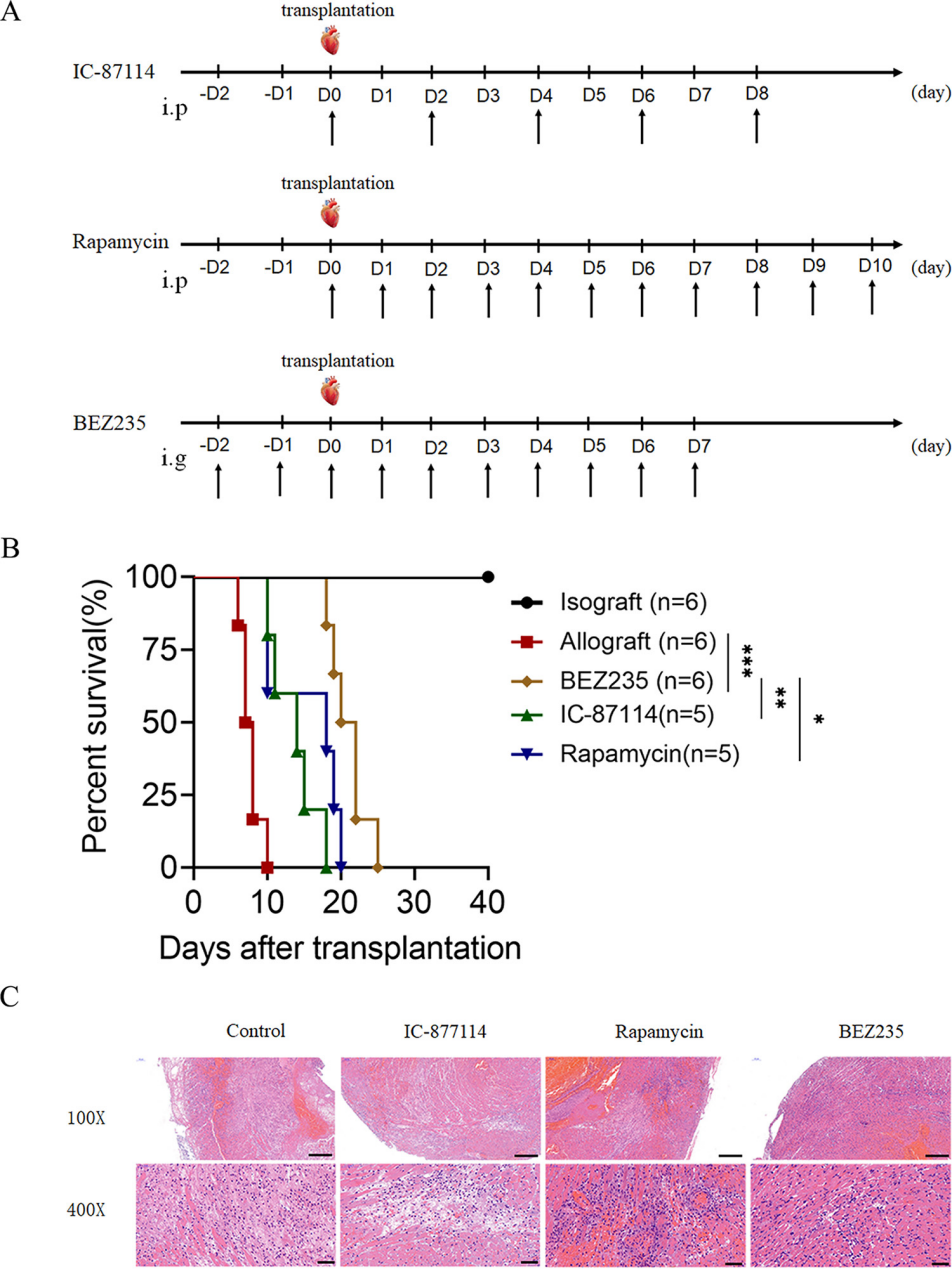
Impact of BEZ235 treatment on allograft survival. (A) IC-87114 (15 mg/kg/day, intraperitoneal injection), rapamycin (2 mg/kg/day, intraperitoneal injection), and BEZ235 (15 mg/kg/day, intragastric administration) were administered to the recipient mice at the indicated times. (B) Survival of the cardiac allografts in the mice. (C) Cardiac allografts were harvested on day 7 after transplantation, and the tissue sections were stained with hematoxylin and eosin (×100 or ×400). The data are shown as the mean ± SEM (*, *P* < 0.05; **, *P* < 0.01; ***, *P* < 0.001).

To investigate the effect of BEZ235 on allograft survival, we established mouse models of allogeneic heterotopic heart transplantation. As shown in Fig. 1A, we established three groups, the IC-87114 (15 mg/kg/day, intraperitoneal injection), rapamycin (2 mg/kg/day, intraperitoneal injection), and BEZ235 (15 mg/kg/day, intragastric administration) groups. We found that treatment with IC-87114 and rapamycin prolonged allograft survival in the corresponding treatment groups compared with that in the allograft group to some extent. Compared to the corresponding treatments (IC-87114 and rapamycin), BEZ235 more efficiently prolonged cardiac transplant survival. BEZ235 treatment significantly prolonged allograft survival compared with that with allograft treatment (mean survival time [MST], 21.0 ± 2.530 versus 7.667 ± 1.366 days, *P* = 0.0006) (Fig. 1B). Hematoxylin and eosin (H&E) staining revealed that the allografts from the three groups displayed reduced inflammatory infiltration in the myocardium compared with that of the control group (Fig. 1C). Of these interventions, BEZ235 treatment significantly alleviated acute rejection in the corresponding BEZ235-treated groups compared with that in the IC-87114 and rapamycin groups on day 7 posttransplantation (Fig. 1C).

### BEZ235 disturbed the intestinal microbiome.

**FIG 2:**
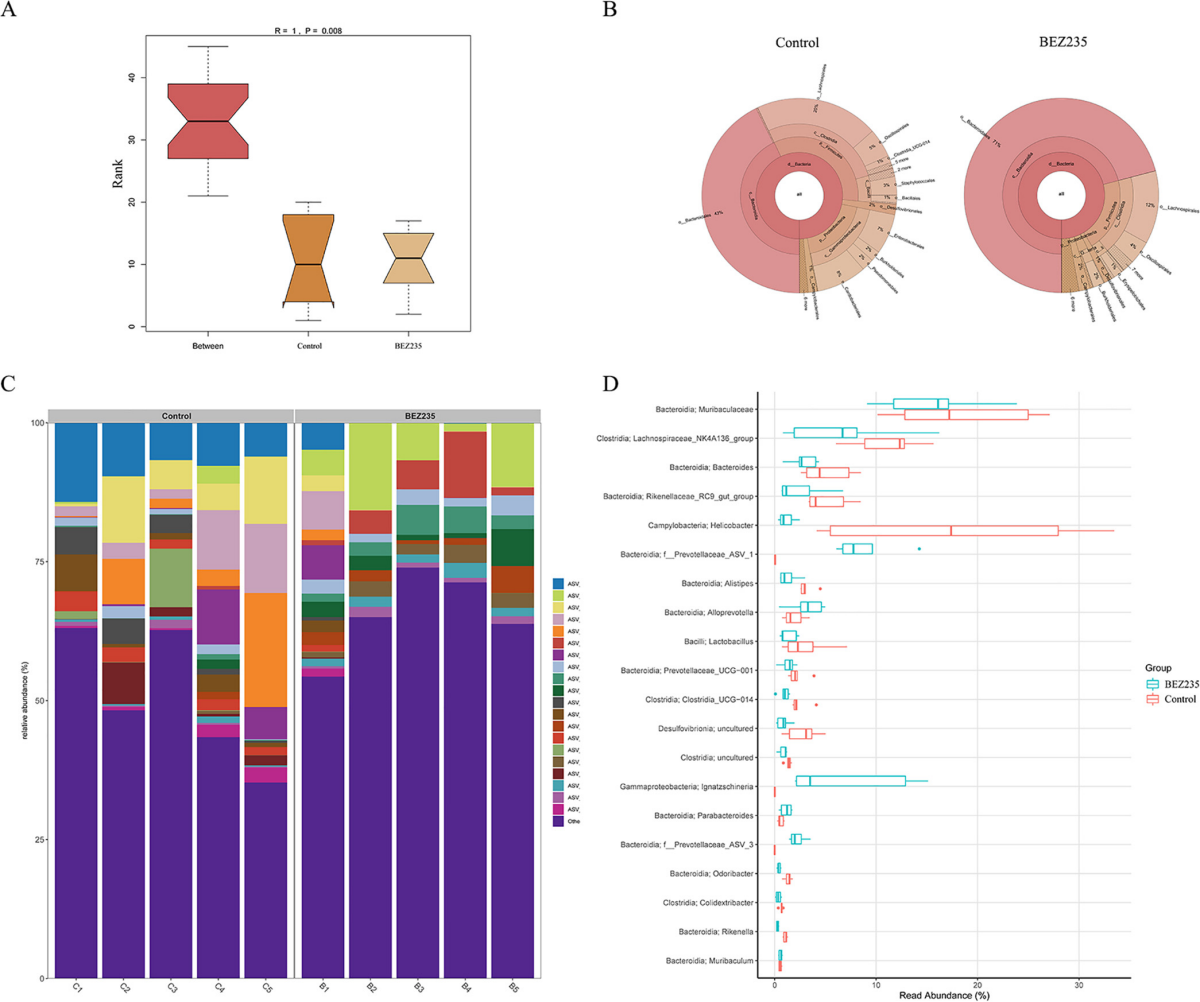
BEZ235 disturbed the intestinal microbiome. (A) Analysis of similarity (ANOSIM). Intergroup differences were compared through ANOSIM. (B) Krona chart. Graphical displays were visualized using a Krona chart. (C and D) Relative abundances of bacteria in the gut microbial community in all samples at the genus level.

We next characterized the alteration of the 16S rRNA gene sequences in fecal samples to assess the influences of BEZ235 on the intestinal microbiome. Analysis of similarity (ANOSIM) showed a significant difference between the BEZ235 group and the control group (ANOSIM, *R* = 1, *P* = 0.008) (Fig. 2A). Figure 2B depicts a Krona visualization of the types and abundances of all bacteria in the two groups. Compared to the control group, the BEZ235 group had significantly lower bacterial richness and diversity. The bar graph and differential abundance analysis were conducted to compare microbe abundance. In the BEZ235-treated mice, the relative abundances of the bacterial species were significantly decreased compared with those in the control mice (Fig. 2C and D).

### Beneficial influence of probiotic supplementation on the intestinal microbiome in BEZ235-treated mice.

**FIG 3:**
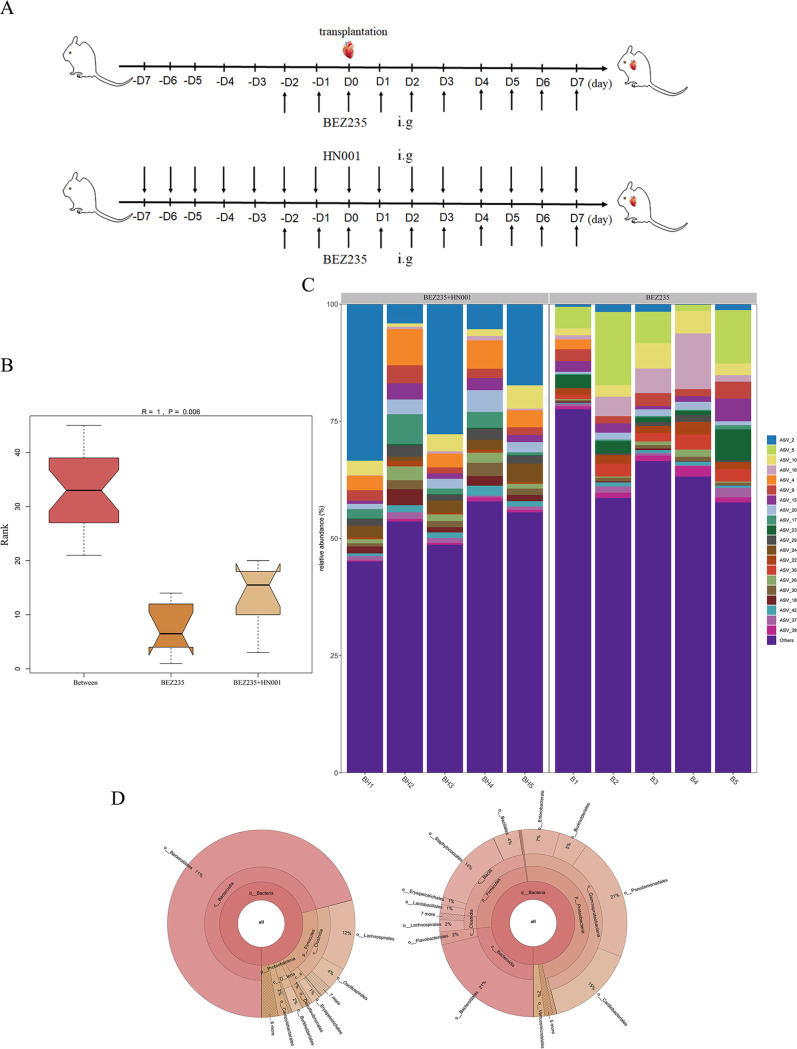
Beneficial influence of probiotic supplementation on the intestinal microbiome in BEZ235-treated mice. (A) Treatment plan for BEZ235 (15 mg/kg/day, intragastric administration) and Lactobacillus rhamnosus HN001 (2 × 10^8^ CFU, intragastric administration). (B) Analysis of similarity (ANOSIM). Intergroup differences were compared through ANOSIM. (C) Relative abundances of the bacteria in the gut microbial community in all samples at the genus level. (D) Krona chart. Graphical displays were visualized using a Krona chart.

We supplemented BEZ235-treated mice with the probiotic Lactobacillus rhamnosus HN001 and observed the relative abundances of the bacterial species. The mice were treated by oral gavage with HN001 twice per day. The treatment plan is shown in Fig. 3A.

ANOSIM showed a significant compositional difference between the BEZ235 group and the combined group (*R* = 1, *P* = 0.006) (Fig. 3B). Differential abundance analysis was conducted to identify the microbe abundance. The relative abundances of bacteria increased in the combined group and were restored to the levels in the control group mice (Fig. 3C). This phenomenon was additionally confirmed by Krona plot analysis (Fig. 3D).

### The combination of BEZ235 and Lactobacillus rhamnosus HN001 attenuated allograft rejection.

**FIG 4:**
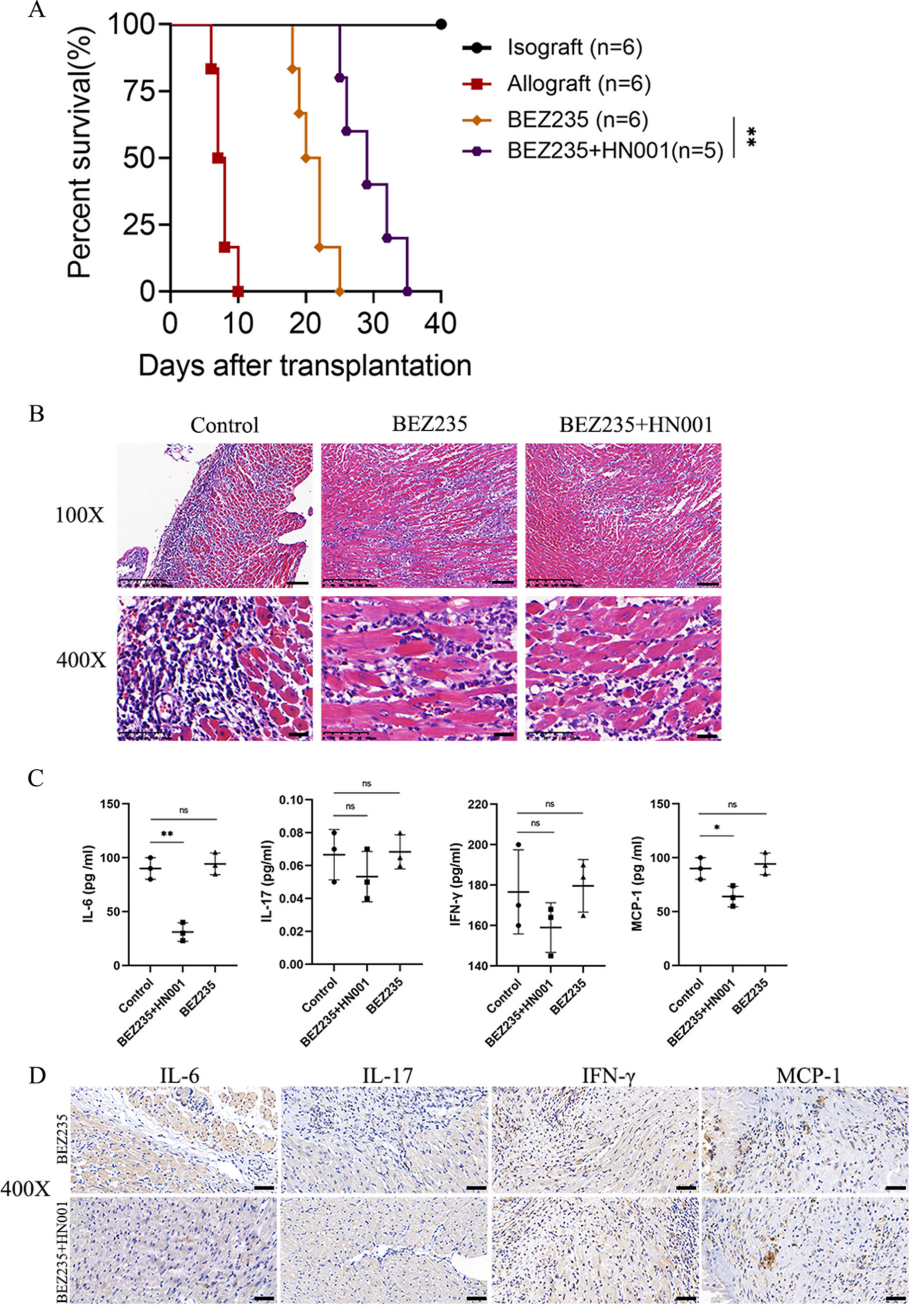
Combination of BEZ235 and Lactobacillus rhamnosus HN001-attenuated allograft rejection. (A) Survival of the cardiac allografts in mice. (B) Cardiac allografts were harvested on day 7 after transplantation, and the tissue sections were stained with hematoxylin and eosin (×100 or ×400). (C) Serum proinflammatory cytokine levels. (D) IL-6, IL-17, MCP-1, and IFN-γ staining of grafts from the BEZ235 treatment group and control group mice at day 7 after heart transplantation (×400 original magnification). Data are shown as the mean ± SEM (ns, *P* ≥ 0.05; *, *P* < 0.05; **, *P* < 0.01).

To determine whether Lactobacillus rhamnosus HN001 influences allograft rejection in the mouse heart transplant model, we supplemented BEZ235-treated mice with the probiotic Lactobacillus rhamnosus HN001 to observe the survival time of cardiac allografts. Interestingly, the combination of BEZ235 and HN001 significantly prolonged allograft survival compared with survival with BEZ235 treatment alone (MST, 29.4 ± 4.159 versus 21.0 ± 2.530 days, *P* = 0.0034) (Fig. 4A). H&E staining showed that the number of lymphocytes infiltrating the grafts was reduced in the probiotic administration group on day 7 posttransplantation (Fig. 4B). We also wanted to determine whether Lactobacillus rhamnosus HN001-attenuated allograft rejection was associated with decreased levels of proinflammatory cytokines. We next determined the expression of proinflammatory cytokines in serum and transplanted cardiac tissues. Probiotic supplementation significantly decreased the levels of serum inflammatory cytokines such as IL-6 and MCP-1 in BEZ235-treated mice (Fig. 4C). However, in the transplanted cardiac tissues of the two groups, the induction of the inflammatory cytokine (IL-6) production was significantly decreased, while that of the other cytokines was not changed (Fig. 4D).

### Metabolomic investigation of probiotic supplementation in the intestinal microbiome.

**FIG 5:**
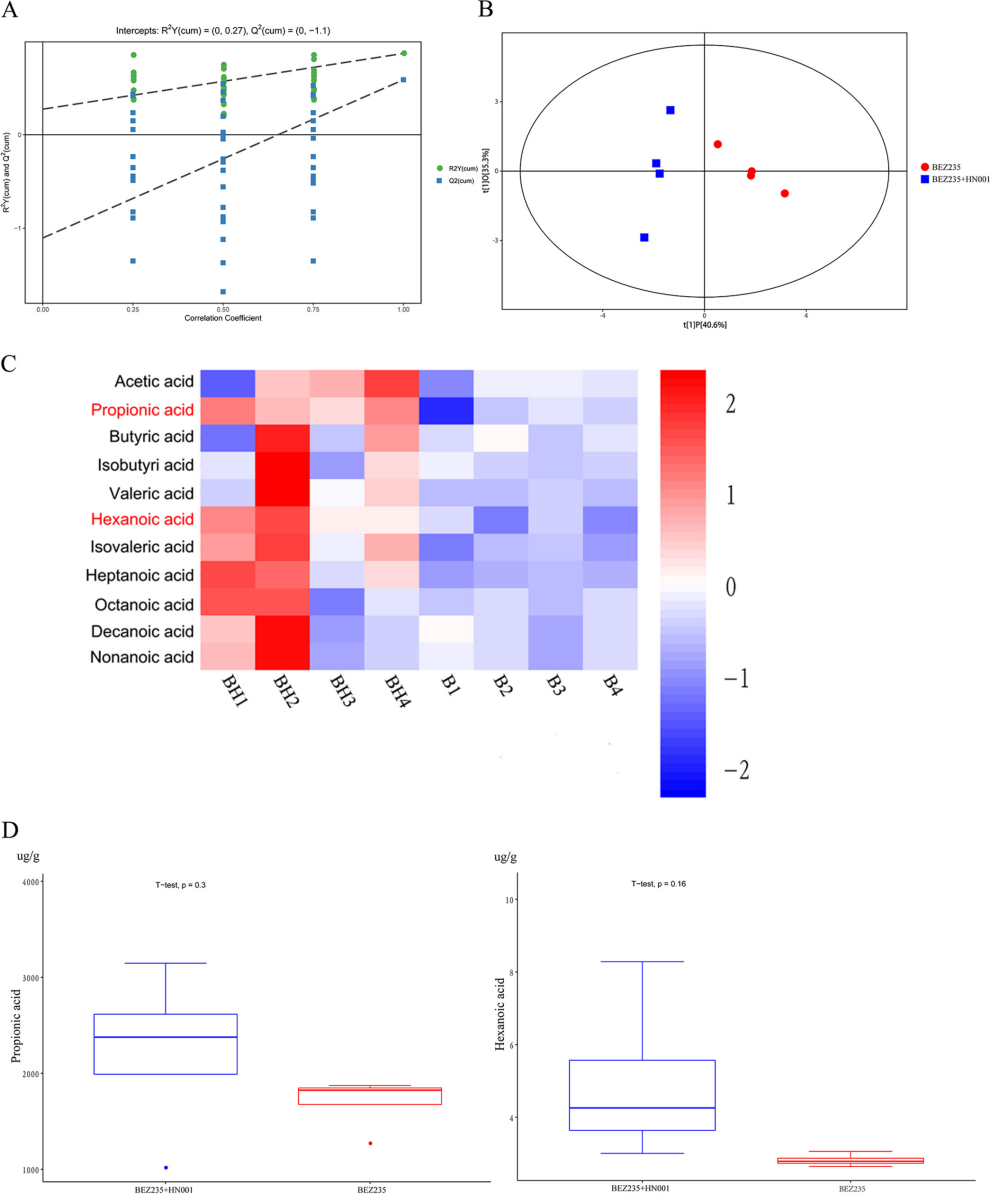
Metabolomic investigation of the effects of probiotic supplementation on the intestinal microbiome. (A) Orthogonal projections to latent structures-discriminant analysis (OPLS-DA) model overview. OPLS-DA was performed for multivariate group analysis. (B) Principal-coordinate analysis (PCoA) of the gut microbiome composition at the genus level based on the weighted UniFrac matrix for each group. The colors of the spots indicate samples from each individual. (C) Hierarchical clustering heatmap of differential metabolite expression between the probiotic administration group and the BEZ235-treated group. (D) Boxplot analysis was used to present the data distribution and to detect outliers. The data are shown as the mean ± SEM (*, *P* < 0.05; **, *P* < 0.01; ***, *P* < 0.001).

Given that we found that probiotic administration was able to attenuate allograft rejection, we next explored the possible molecular mechanisms underlying the beneficial effects of Lactobacillus rhamnosus HN001. As shown in Fig. 5A, the orthogonal projections to latent structures-discriminant analysis (OPLS-DA) replacement test showed that the original model had good robustness and no overfitting issues. A score plot of the OPLS-DA comparing the metabolic differences revealed a clear segregation between the probiotic administration group and the BEZ235-treated group (Fig. 5B). We next used gas chromatography-mass spectrometry (GC-MS) to detect the concentrations of fecal SCFAs on day 7 posttransplantation and found that PA and hexanoic acid (HA) were significantly more abundant in the probiotic administration group than in the BEZ235-treated group (Fig. 5C). The levels of other metabolites did not show statistically significant differences. Boxplot analysis also confirmed this phenomenon (Fig. 5D). These data indicated that Lactobacillus rhamnosus HN001 may exert its effects through PA and HA.

### Propionic acid, not hexanoic acid, prolonged transplant survival.

**FIG 6:**
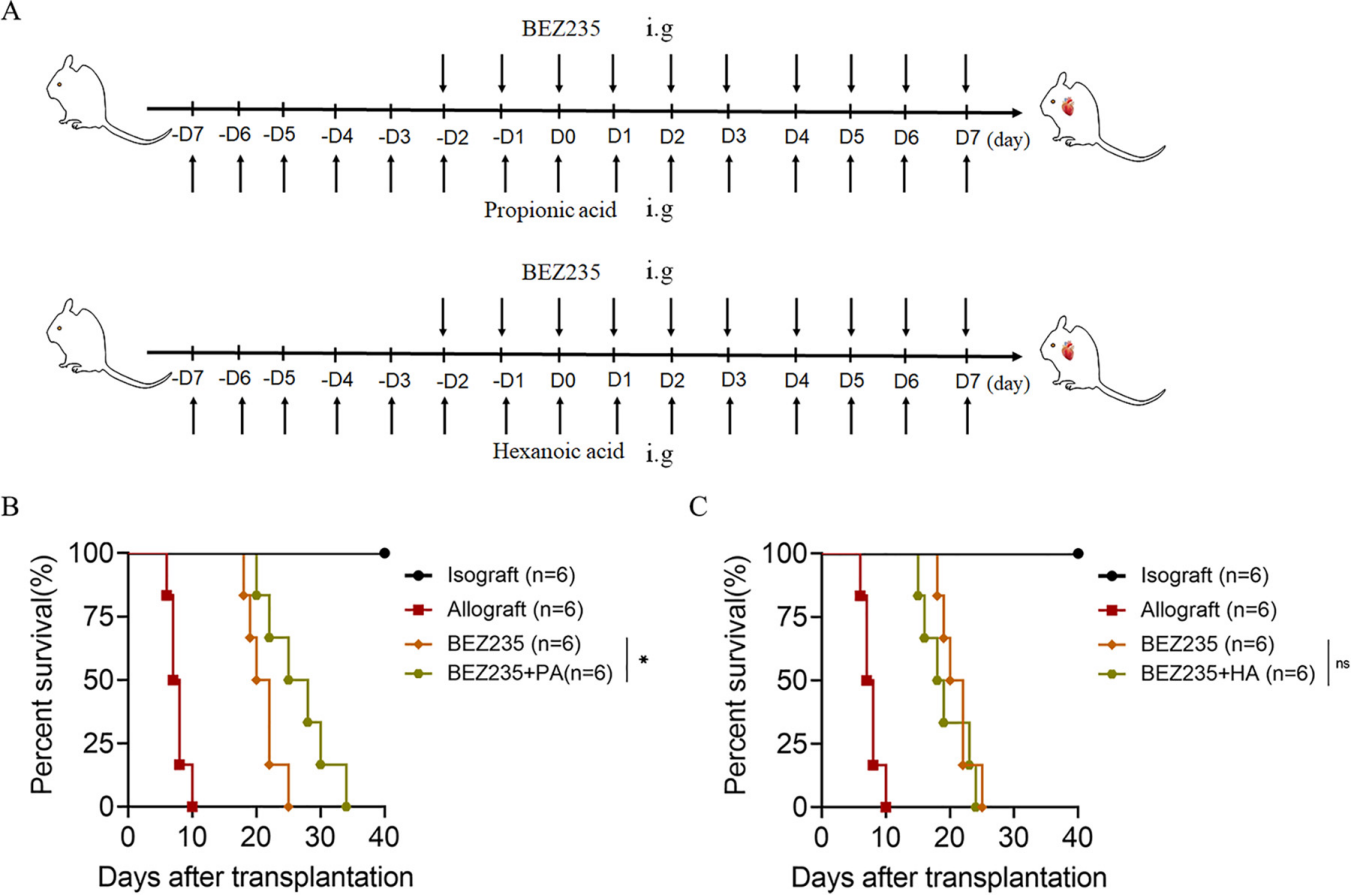
Propionic acid, not hexanoic acid, prolonged transplant survival. (A) Treatment plan of PA (500 mg twice daily, intragastric administration) and HA (500 mg twice daily, intragastric administration). (B and C) Survival of the cardiac allografts in the mice. The data are shown as the mean ± SEM (ns, *P* ≥ 0.05; *, *P* < 0.05; **, *P* < 0.01).

We further investigated which metabolite was required for Lactobacillus rhamnosus HN001 to exert its effects. We supplemented BEZ235-treated mice with 500 mg of PA or HA twice daily over the course of the experiment. The treatment plan is shown in Fig. 6A. The survival time of cardiac allografts in the two groups was calculated. The PA supplementation group had prolonged allograft survival compared with that of the BEZ235-treated group (MST, 25.0 ± 4.123 versus 21.0 ± 2.530 days, *P* = 0.0351) (Fig. 6B). No significant difference was observed in allograft survival times between the HA supplementation group and the BEZ235-treated group (Fig. 6C). These results indicated that PA was required for Lactobacillus rhamnosus HN001 to attenuate allograft rejection.

### Propionic acid supplementation enhanced the number and suppressive function of regulatory T cells in BEZ235-treated mice.

**FIG 7:**
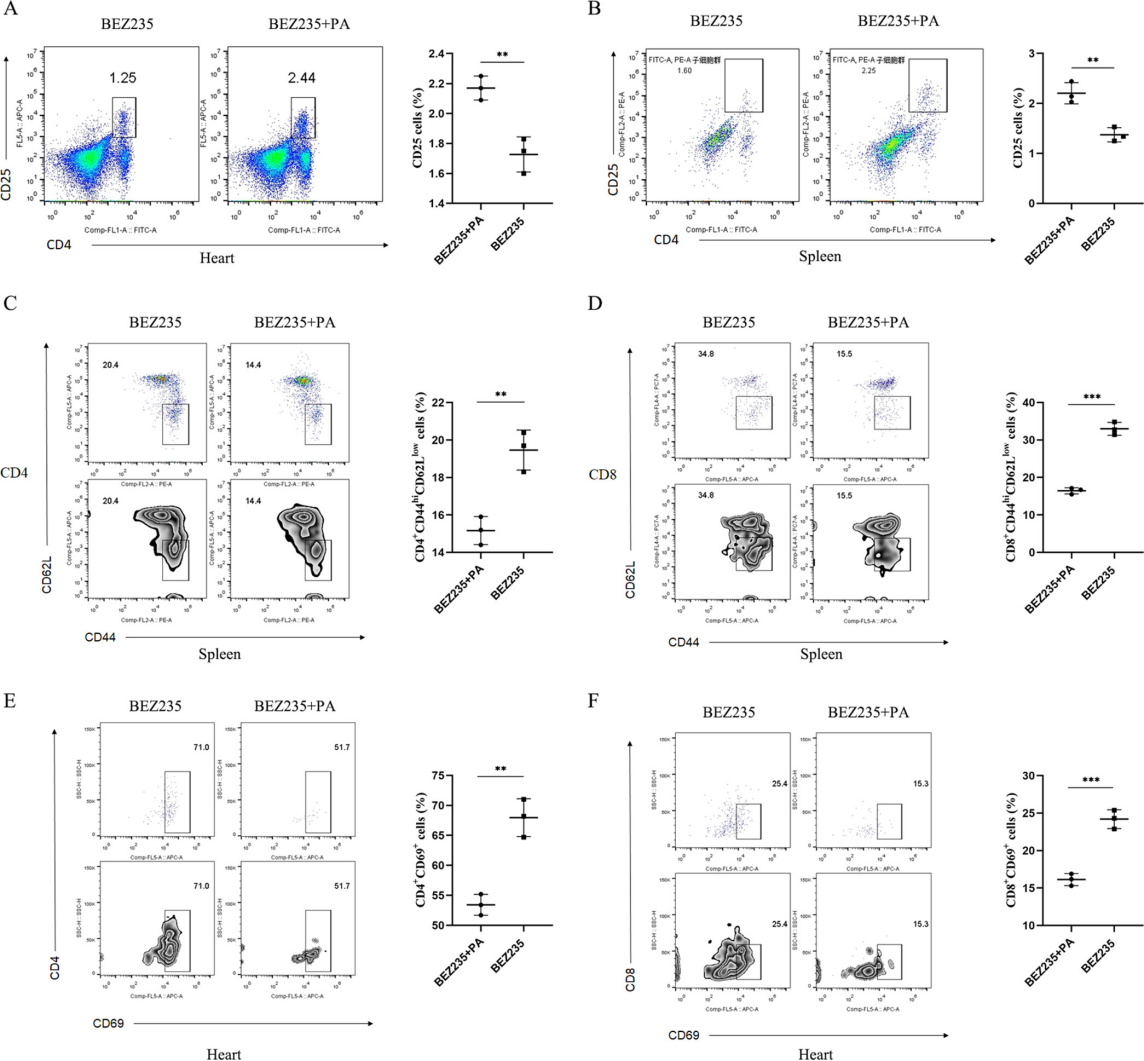
Propionic acid supplementation enhanced the number and suppressive function of regulatory T cells in BEZ235-treated mice. (A and B) Representative flow cytometry plots showing an enriched Treg population in the propionic acid supplementation group in comparison to that in the BEZ235-treated group at day 7 after heart transplantation. Heart (left) and splenocytes (right). (C and D) Propionic acid-supplemented mice showed a lower frequency of CD4^+^ T effector cells (left) and CD8^+^ effector cells (right). (E and F) CD69 expression in graft-infiltrating CD4^+^ T cells and CD8^+^ T cells from the two treatment groups (BEZ235, BEZ235+PA) on day 7 posttransplantation. The data are shown as the mean ± SEM (**, *P* < 0.01; ***, *P* < 0.001).

Given current insights into the modulatory effects of PA on the immune system (31–33), we next assessed the impact of PA on the proportion of immune cells in heterotopic cardiac graft tissues and spleen tissues after cardiac transplantation. We found that there was a significantly higher proportion of Treg cells (CD4^+^ CD25^+^) in heterotopic cardiac graft tissues and spleen tissues isolated from PA-supplemented mice on day 7 posttransplantation than in those from BEZ235-treated control mice (Fig. 7A and B). Next, we examined Treg suppressive function with PA supplementation. The percentage of T effector cells (CD44^hi^ CD62L^low^) among splenic CD4^+^ and CD8^+^ T cells was significantly lower in the PA supplementation group than in the BEZ235-treated control group (Fig. 7C and D). Intragraft CD4^+^ and CD8^+^ T cells from PA-supplemented mice showed lower expression of the activation marker CD69 than those from BEZ235-treated mice (Fig. 7E and F). Taken together, these analyses suggested that PA supplementation enhanced the frequency and function of Treg cells in BEZ235-treated mice.

### Effect of PA supplementation on the neuron/IL-6 axis.

**FIG 8:**
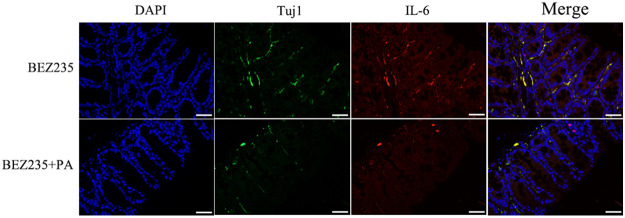
Effect of propionic acid supplementation on the neuron/IL-6 axis. Immunofluorescence assay showing the expression of Tuj1 and IL-6 in intestinal tissues derived from the two treatment groups (BEZ235, BEZ235+PA).

There is some evidence that IL-6 is released by enteric neurons (34). In the present study, we hypothesized that intestinal microbial metabolites affect the release of IL-6 from enteric neurons. Immunofluorescent imaging was performed on colons from PA-supplemented mice (nerve fibers were identified by anti-Tuj1 antibody staining). As we anticipated, PA supplementation inhibited IL-6 release by enteric neurons (Fig. 8A).

## DISCUSSION

The results presented in this study demonstrate that low-dose PI3K/mTOR dual inhibition (BEZ235) significantly prolongs murine cardiac allograft survival. Moreover, we found that the low dose of BEZ235 disturbed intestinal homeostasis. The beneficial effects of probiotic supplementation were observed in BEZ235-induced mouse models with IM disturbance. Apart from the effects of regulating the IM and restoring homeostasis, beneficial immunomodulatory effects have also been observed following probiotic supplementation. Probiotics may serve as potent immunomodulatory supplements to BEZ235 therapy. These findings provide a novel and efficient therapeutic strategy for treating transplant recipients.

The effects of mTOR inhibitors on host intestinal homeostasis have been reported. As a commercially available probiotic, Lactobacillus rhamnosus HN001 is known to regulate the IM (26). To overcome the IM disturbance in BEZ235-treated mice, we selected Lactobacillus rhamnosus HN001 as a potential regulator. Lactobacillus rhamnosus HN001 is well known for its health-promoting properties in the gut (20–22, 25) and has been widely used in the food industry as a probiotic therapy for children with acute infectious diarrhea (35). Consistent with these findings, we observed that Lactobacillus rhamnosus HN001 markedly protected against disorders of the IM in BEZ235-treated mice.

Although progress has been achieved in the treatment of allograft rejection, the efficacy is not very satisfactory. Our data demonstrated that supplementation of BEZ235-treated mice with the probiotic Lactobacillus rhamnosus HN001 significantly prolonged allograft survival. However, the underlying mechanism by which probiotic supplementation reduces allograft rejection remains unclear. Gut metabolites, which are jointly generated by the gut microbiota, are important modulators of host physiologic functions and metabolism (36–38). SCFAs are important metabolites produced by intestinal bacteria and mediate beneficial effects on host metabolic health (28, 39). Our data indicated that PA levels changed during probiotic supplementation and that PA was required for Lactobacillus rhamnosus HN001 to exert its function. Recent experimental studies have indicated that PA could protect against hypertensive and ischemic cardiac injury by maintaining immune homeostasis (40). PA normalizes Treg cell mitochondrial function and serves as a potent immunomodulatory supplement to multiple sclerosis drugs (30). In our model of allogeneic heterotopic heart transplantation, PA supplementation led to a simultaneous increase in both the proportion and function of Tregs. It has been documented that microbial signals promote Treg cell generation via neuron-produced IL-6 (34). Therefore, our findings reveal a novel regulatory mechanism. As a metabolite of Lactobacillus rhamnosus HN001, PA increases both the proportion and function of Tregs via neuron-produced IL-6.

Relatively long-term graft survival remains a major concern in the transplantation field. We demonstrated that Lactobacillus rhamnosus HN001 combined with BEZ235 prolonged murine cardiac allograft survival to some extent. The therapeutic function of BEZ235, which inhibits PI3K (p110-α, -β, -γ, and -δ isoforms) and mTOR kinase activity by binding to the ATP-binding cleft of these enzymes, has been widely demonstrated in various cancers (41–43). The results from our experiments suggest that inclusion of a low dose of BEZ235 with transplantation could enhance the efficacy of treatment. There are concerns about its detrimental effect on heart transplant recipients, which is thought to be due to its competitive inhibition of PI3K and mTOR. However, with the low dose of BEZ235, no severe adverse side effects were found in our animal studies. Given the significant survival advantage seen in our combination therapy-treated mice, a combined therapy option for transplant recipients is likely possible.

Our study demonstrated that probiotic supplementation is critical for the fine-tuning of the immune response. However, a limitation of our study is that probiotic supplementation is potentially causative of BEZ235-induced metabolic disorders in transplant recipient mice. In our future investigations, antibiotic-treated or germfree mice will need to be applied to clarify the connections between the gut microbiota and the immune system in the context of transplantation. Furthermore, the availability of probiotic supplementation suggests that this approach could be efficiently translated into novel and effective strategies for the prevention of graft rejection.

## MATERIALS AND METHODS

### Animals and experimental design.

Male C57BL/6 (B6; H-2^b^) and BALB/c (B/c; H-2^d^) mice (8 weeks old) were purchased from Beijing Vital River Laboratory Animal Technology Co., Ltd. (Beijing, China). The animal experiment was approved by the Institutional Animal Care and Use Committee of Zhejiang University. To balance the IM among mice, all mice were housed under specific-pathogen-free conditions on a 12-h light/12-h dark cycle with freely available food and water.

IC-87114 (catalog no. S1268; Selleckchem) was injected intraperitoneally (i.p.) at 15 mg/kg for five doses into the recipients at the indicated times. Rapamycin (catalog no. S1039; Selleckchem) was administered at the indicated times (2 mg/kg on days 0 to 10). BEZ235 (catalog no. S1009; Selleckchem) was orally administered via gavage at 15 mg/kg on the indicated dates. Group allocation for the experiments was randomized and not blinded. BEZ235-treated mice were administered PA (500 mg, intragastric administration) or HA (500 mg, intragastric administration) by gavage twice per day for 2 weeks.

In the probiotic experiment, the mice were randomly assigned to the control group (*n* = 6), BEZ235 group (*n* = 5), and probiotics group (BEZ235 and 2 × 10^8^ CFU of Lactobacillus rhamnosus HN001; *n* = 6). Lactobacillus rhamnosus HN001 (provided by Nutrition & Biosciences, DuPont) was resuspended in 200 μL of phosphate-buffered saline and orally administered to the BEZ235-treated mice by gavage twice per day for 2 weeks.

Animals were euthanized by dissecting the diaphragm under isoflurane anesthesia, after which organs were harvested. The sacrifice respected the Declaration of Helsinki's rules. All experiments were conducted in accordance with the guidelines of the National Institutes of Health Guide for the Care and Use of Laboratory Animals, and the study was approved by the Animal Care and Use Committee of the Second Affiliated Hospital, School of Medicine, Zhejiang University (2020-109).

### Vascularized heterotopic cardiac transplantation.

The vascularized heterotopic heart transplantation model was established as previously described (44). Briefly, hearts harvested from male C57BL/6 donors (B6; H-2^b^) were transplanted into the subcutaneous space of the right neck of male BALB/c recipient mice (B/c; H-2^d^). Cardiac grafts, peripheral blood, and spleens were harvested.

### ELISA.

Mouse interferon gamma (IFN-γ), IL-6, IL-17, and MCP-1 enzyme-linked immunosorbent assay (ELISA) kits (BioLegend; ELISA Max deluxe sets) were used for the analysis of cytokines in mouse serum. Final calculations were based on a regression analysis of the log of the final optical density versus the log of standard dilutions, and concentrations were reported in picograms per milliliter.

### 16S rRNA sequencing of the intestinal microbiota.

Bacterial genomic DNA was extracted from samples using a FastDNA Spin kit for soil according to the manuals. Bacterial diversity was characterized via amplification of a PCR product that was excised from a 1.5% agarose gel, recovered using GeneClean Turbo (MP Biomedicals), and quantified by a Quant-iT PicoGreen double-stranded DNA (dsDNA) assay kit (Life Technologies, Grand Island, NY). The V3-V4 region of the bacterial 16S rRNA gene was amplified by PCR using a multiplex approach with a primer pair. Prior to sequencing, the amount of input DNA per sample was normalized using a SequalPrep normalization plate following the standard protocol (Thermo Fisher Scientific, Wilmington, DE, USA).

### Extraction and quantification of the gut microbiota metabolites in feces.

Briefly, 10 mg of fecal sample was homogenized using 2.8-mm Precellys ceramic beads (Bertin Technologies) with an extraction solution containing 100 mL of 100 mM crotonic acid (Sigma) as an internal standard, 50 mL of HCl (Sigma), and 200 mL of ether (Sigma). Samples were homogenized for 10 min at room temperature at 1,500 rpm on a shaker (Eppendorf), followed by 10 min of centrifugation at 1,000 × *g*. From the upper ether phase, 80 mL was transferred into a new glass vial containing 16 mL of *N*-*tert*-butyldimethylsilyl-*N*-methyltrifluoroacetamide (MTBSTFA; Sigma). The analytes were detected using the full scan mode. The concentration of SCFAs was calculated using the external standard method and expressed as micromole per gram sample.

### Histopathology.

Cardiac grafts were harvested 5 and 7 days after transplantation. Samples were transversely sliced and fixed in 10% formalin (catalog no. SF98-4; Fisher) at 4°C until use. The fixed tissues were dehydrated and processed for paraffin embedding, and 5-μm sections were stained with H&E.

### Immunohistochemical (IHC) staining and immunofluorescence.

Briefly, sections were incubated with 0.3% hydrogen peroxide in methanol for 20 min to inhibit endogenous peroxidase activity and heated in citrate buffer at 121°C for 30 min to determine antigenic activity. The slides were incubated with rabbit polyclonal antibodies at 4°C overnight, followed by incubation with an Alexa Fluor-conjugated secondary antibody (Life Technologies) diluted in blocking buffer for 1 h at room temperature. Slides were examined by using a laser scanning confocal microscope (Zeiss LSM 800).

### Cell isolation and flow cytometric analysis.

The grafts were incubated in RPMI 1640 medium containing 0.5 mg/mL collagenase IV (Sigma-Aldrich, St. Louis, MO) for 30 min at 37°C. Tissue debris was removed by a 70-μm cell strainer. After washing twice, viable mononuclear cells were separated by Ficoll density gradient centrifugation. The cells were washed three times with RPMI 1640 medium supplemented with 10% fetal bovine serum (FBS) and then subjected to fluorescence-activated cell sorting (FACS) staining. They were blocked, stained, and analyzed using FACSCalibur (BD) and FlowJo software (Tree Star).

### Statistical analysis.

SPSS v23 (SPSS Inc., Chicago, IL) was used for experimental data analysis, and data passed normality and equal variance tests. All experiments were independently repeated at least three times. The sample size was calculated by using PASS 11 (NCSS Inc.). Statistical comparisons between 2 groups involved Student's *t* test and, otherwise, one-way analysis of variance (ANOVA) and Bonferroni posttests. All data are expressed as the mean ± standard error of the mean (SEM). All statistical tests were two-tailed, and *P* values of <0.05 were considered statistically significant.

### Data availability.

All data generated in the study are included in this article. Raw sequence data are accessible on the NCBI platform under accession no. PRJNA836559.

## References

[1] Stehlik J, Edwards LB, Kucheryavaya AY, Aurora P, Christie JD, Kirk R, Dobbels F, Rahmel AO, Hertz MI. 2010. The Registry of the International Society for Heart and Lung Transplantation: twenty-seventh official adult heart transplant report–2010. J Heart Lung Transplant 29:1089–1103. doi:10.1016/j.healun.2010.08.007. 20870164

[2] Yang C, Chen X, Wei Z, Xiao J, Chen W, Shang Y, Liu J. 2018. Targeting the class IA PI3K isoforms p110alpha/delta attenuates heart allograft rejection in mice by suppressing the CD4(+) T lymphocyte response. Am J Transl Res 10:1387–1399. 29887953 PMC5992545

[3] Shin HJ, Baker J, Leveson-Gower DB, Smith AT, Sega EI, Negrin RS. 2011. Rapamycin and IL-2 reduce lethal acute graft-versus-host disease associated with increased expansion of donor type CD4+CD25+Foxp3+ regulatory T cells. Blood 118:2342–2350. doi:10.1182/blood-2010-10-313684. 21734238 PMC4467868

[4] Okkenhaug K, Vanhaesebroeck B. 2003. PI3K in lymphocyte development, differentiation and activation. Nat Rev Immunol 3:317–330. doi:10.1038/nri1056. 12669022

[5] Okkenhaug K, Patton DT, Bilancio A, Garcon F, Rowan WC, Vanhaesebroeck B. 2006. The p110 delta isoform of phosphoinositide 3-kinase controls clonal expansion and differentiation of Th cells. J Immunol 177:5122–5128. doi:10.4049/jimmunol.177.8.5122. 17015696

[6] Deng L, Jiang L, Lin X-H, Tseng K-F, Liu Y, Zhang X, Dong R-H, Lu Z-G, Wang X-J. 2017. The PI3K/mTOR dual inhibitor BEZ235 suppresses proliferation and migration and reverses multidrug resistance in acute myeloid leukemia. Acta Pharmacol Sin 38:382–391. doi:10.1038/aps.2016.121. 28042875 PMC5342661

[7] Wang Y, Miao X, Jiang Y, Wu Z, Zhu X, Liu H, Wu X, Cai J, Ding X, Gong W. 2022. The synergistic antitumor effect of IL-6 neutralization with NVP-BEZ235 in hepatocellular carcinoma. Cell Death Dis 13:146. doi:10.1038/s41419-022-04583-5. 35165269 PMC8844296

[8] Carrabotta M, Laginestra MA, Durante G, Mancarella C, Landuzzi L, Parra A, Ruzzi F, Toracchio L, De Feo A, Giusti V, Pasello M, Righi A, Lollini PL, Palmerini E, Donati DM, Manara MC, Scotlandi K. 2022. Integrated molecular characterization of patient-derived models reveals therapeutic strategies for treating CIC-DUX4 sarcoma. Cancer Res 82:708–720. doi:10.1158/0008-5472.CAN-21-1222. 34903601 PMC9359717

[9] Ma Y, Jin Z, Yu K, Liu Q. 2019. NVP-BEZ235-induced autophagy as a potential therapeutic approach for multiple myeloma. Am J Transl Res 11:87–105. 30787971 PMC6357299

[10] Herrero-Sánchez MC, Rodríguez-Serrano C, Almeida J, San Segundo L, Inogés S, Santos-Briz Á, García-Briñón J, Corchete LA, San Miguel JF, Del Cañizo C, Blanco B. 2016. Targeting of PI3K/AKT/mTOR pathway to inhibit T cell activation and prevent graft-versus-host disease development. J Hematol Oncol 9:113. doi:10.1186/s13045-016-0343-5. 27765055 PMC5072323

[11] Saxton RA, Sabatini DM. 2017. mTOR signaling in growth, metabolism, and disease. Cell 169:361–371. doi:10.1016/j.cell.2017.03.035. 28388417

[12] Chow J, Lee SM, Shen Y, Khosravi A, Mazmanian SK. 2010. Host-bacterial symbiosis in health and disease. Adv Immunol 107:243–274. doi:10.1016/B978-0-12-381300-8.00008-3. 21034976 PMC3152488

[13] Wang L, Cheng X, Bai L, Gao M, Kang G, Cao X, Huang H. 2022. Positive interventional effect of engineered butyrate-producing bacteria on metabolic disorders and intestinal flora disruption in obese mice. Microbiol Spectr 10:e0114721. doi:10.1128/spectrum.01147-21. 35293806 PMC9045090

[14] Hu Y, Pan Z, Huang Z, Li Y, Han N, Zhuang X, Peng H, Gao Q, Wang Q, Yang Lee BJ, Zhang H, Yang R, Bi Y, Xu ZZ. 2022. Gut microbiome-targeted modulations regulate metabolic profiles and alleviate altitude-related cardiac hypertrophy in rats. Microbiol Spectr 10:e0105321. doi:10.1128/spectrum.01053-21. 35138162 PMC8826942

[15] Postler TS, Ghosh S. 2017. Understanding the holobiont: how microbial metabolites affect human health and shape the immune system. Cell Metab 26:110–130. doi:10.1016/j.cmet.2017.05.008. 28625867 PMC5535818

[16] Hooper LV, Littman DR, Macpherson AJ. 2012. Interactions between the microbiota and the immune system. Science 336:1268–1273. doi:10.1126/science.1223490. 22674334 PMC4420145

[17] McAleer JP, Kolls JK. 2012. Maintaining poise: commensal microbiota calibrate interferon responses. Immunity 37:10–12. doi:10.1016/j.immuni.2012.07.001. 22840839

[18] Suez J, Zmora N, Segal E, Elinav E. 2019. The pros, cons, and many unknowns of probiotics. Nat Med 25:716–729. doi:10.1038/s41591-019-0439-x. 31061539

[19] Qu S, Fan L, Qi Y, Xu C, Hu Y, Chen S, Liu W, Liu W, Si J. 2021. Akkermansia muciniphila alleviates dextran sulfate sodium (DSS)-induced acute colitis by NLRP3 activation. Microbiol Spectr 9:e0073021. doi:10.1128/Spectrum.00730-21. 34612661 PMC8510245

[20] Gill HS, Rutherfurd KJ, Prasad J, Gopal PK. 2000. Enhancement of natural and acquired immunity by Lactobacillus rhamnosus (HN001), Lactobacillus acidophilus (HN017) and Bifidobacterium lactis (HN019). Br J Nutr 83:167–176. doi:10.1017/s0007114500000210. 10743496

[21] Sheih YH, Chiang BL, Wang LH, Liao CK, Gill HS. 2001. Systemic immunity-enhancing effects in healthy subjects following dietary consumption of the lactic acid bacterium Lactobacillus rhamnosus HN001. J Am Coll Nutr 20:149–156. doi:10.1080/07315724.2001.10719027. 11349938

[22] Good M, Sodhi CP, Ozolek JA, Buck RH, Goehring KC, Thomas DL, Vikram A, Bibby K, Morowitz MJ, Firek B, Lu P, Hackam DJ. 2014. Lactobacillus rhamnosus HN001 decreases the severity of necrotizing enterocolitis in neonatal mice and preterm piglets: evidence in mice for a role of TLR9. Am J Physiol Gastrointest Liver Physiol 306:G1021–G1032. doi:10.1152/ajpgi.00452.2013. 24742987 PMC4042115

[23] Anderson RC, Cookson AL, McNabb WC, Kelly WJ, Roy NC. 2010. Lactobacillus plantarum DSM 2648 is a potential probiotic that enhances intestinal barrier function. FEMS Microbiol Lett 309:184–192. doi:10.1111/j.1574-6968.2010.02038.x. 20618863

[24] Toscano M, De Grandi R, Stronati L, Vecchi ED, Drago L. 2017. Effect of Lactobacillus rhamnosus HN001 and Bifidobacterium longum BB536 on the healthy gut microbiota composition at phyla and species level: a preliminary study. World J Gastroenterol 23:2696–2704. doi:10.3748/wjg.v23.i15.2696. 28487606 PMC5403748

[25] Wickens KL, Barthow CA, Murphy R, Abels PR, Maude RM, Stone PR, Mitchell EA, Stanley TV, Purdie GL, Kang JM, Hood FE, Rowden JL, Barnes PK, Fitzharris PF, Crane J. 2017. Early pregnancy probiotic supplementation with Lactobacillus rhamnosus HN001 may reduce the prevalence of gestational diabetes mellitus: a randomised controlled trial. Br J Nutr 117:804–813. doi:10.1017/S0007114517000289. 28367765 PMC5426322

[26] Han Y, Wu L, Ling Q, Wu P, Zhang C, Jia L, Weng H, Wang B. 2021. Intestinal dysbiosis correlates with sirolimus-induced metabolic disorders in mice. Transplantation 105:1017–1029. doi:10.1097/TP.0000000000003494. 33116044

[27] Koh A, De Vadder F, Kovatcheva-Datchary P, Backhed F. 2016. From dietary fiber to host physiology: short-chain fatty acids as key bacterial metabolites. Cell 165:1332–1345. doi:10.1016/j.cell.2016.05.041. 27259147

[28] Kaye DM, Shihata WA, Jama HA, Tsyganov K, Ziemann M, Kiriazis H, Horlock D, Vijay A, Giam B, Vinh A, Johnson C, Fiedler A, Donner D, Snelson M, Coughlan MT, Phillips S, Du X-J, El-Osta A, Drummond G, Lambert GW, Spector TD, Valdes AM, Mackay CR, Marques FZ. 2020. Deficiency of prebiotic fiber and insufficient signaling through gut metabolite-sensing receptors leads to cardiovascular disease. Circulation 141:1393–1403. doi:10.1161/CIRCULATIONAHA.119.043081. 32093510

[29] Smith PM, Howitt MR, Panikov N, Michaud M, Gallini CA, Bohlooly-Y M, Glickman JN, Garrett WS. 2013. The microbial metabolites, short-chain fatty acids, regulate colonic Treg cell homeostasis. Science 341:569–573. doi:10.1126/science.1241165. 23828891 PMC3807819

[30] Duscha A, Gisevius B, Hirschberg S, Yissachar N, Stangl GI, Eilers E, Bader V, Haase S, Kaisler J, David C, Schneider R, Troisi R, Zent D, Hegelmaier T, Dokalis N, Gerstein S, Del Mare-Roumani S, Amidror S, Staszewski O, Poschmann G, Stühler K, Hirche F, Balogh A, Kempa S, Träger P, Zaiss MM, Holm JB, Massa MG, Nielsen HB, Faissner A, Lukas C, Gatermann SG, Scholz M, Przuntek H, Prinz M, Forslund SK, Winklhofer KF, Müller DN, Linker RA, Gold R, Haghikia A. 2020. Propionic acid shapes the multiple sclerosis disease course by an immunomodulatory mechanism. Cell 180:1067–1080.e16. doi:10.1016/j.cell.2020.02.035. 32160527

[31] Haghikia A, Jörg S, Duscha A, Berg J, Manzel A, Waschbisch A, Hammer A, Lee D-H, May C, Wilck N, Balogh A, Ostermann AI, Schebb NH, Akkad DA, Grohme DA, Kleinewietfeld M, Kempa S, Thöne J, Demir S, Müller DN, Gold R, Linker RA. 2015. Dietary fatty acids directly impact central nervous system autoimmunity via the small intestine. Immunity 43:817–829. doi:10.1016/j.immuni.2015.09.007. 26488817

[32] Haghikia A, Zimmermann F, Schumann P, Jasina A, Roessler J, Schmidt D, Heinze P, Kaisler J, Nageswaran V, Aigner A, Ceglarek U, Cineus R, Hegazy AN, van der Vorst EPC, Döring Y, Strauch CM, Nemet I, Tremaroli V, Dwibedi C, Kränkel N, Leistner DM, Heimesaat MM, Bereswill S, Rauch G, Seeland U, Soehnlein O, Müller DN, Gold R, Bäckhed F, Hazen SL, Haghikia A, Landmesser U. 2022. Propionate attenuates atherosclerosis by immune-dependent regulation of intestinal cholesterol metabolism. Eur Heart J 43:518–533. doi:10.1093/eurheartj/ehab644. 34597388 PMC9097250

[33] Arpaia N, Campbell C, Fan X, Dikiy S, van der Veeken J, deRoos P, Liu H, Cross JR, Pfeffer K, Coffer PJ, Rudensky AY. 2013. Metabolites produced by commensal bacteria promote peripheral regulatory T-cell generation. Nature 504:451–455. doi:10.1038/nature12726. 24226773 PMC3869884

[34] Yan Y, Ramanan D, Rozenberg M, McGovern K, Rastelli D, Vijaykumar B, Yaghi O, Voisin T, Mosaheb M, Chiu I, Itzkovitz S, Rao M, Mathis D, Benoist C. 2021. Interleukin-6 produced by enteric neurons regulates the number and phenotype of microbe-responsive regulatory T cells in the gut. Immunity 54:499–513.e5. doi:10.1016/j.immuni.2021.02.002. 33691135 PMC8133394

[35] Van Niel CW, Feudtner C, Garrison MM, Christakis DA. 2002. Lactobacillus therapy for acute infectious diarrhea in children: a meta-analysis. Pediatrics 109:678–684. doi:10.1542/peds.109.4.678. 11927715

[36] Tang WHW, Backhed F, Landmesser U, Hazen SL. 2019. Intestinal microbiota in cardiovascular health and disease: JACC state-of-the-art review. J Am Coll Cardiol 73:2089–2105. doi:10.1016/j.jacc.2019.03.024. 31023434 PMC6518422

[37] Tang WH, Kitai T, Hazen SL. 2017. Gut microbiota in cardiovascular health and disease. Circ Res 120:1183–1196. doi:10.1161/CIRCRESAHA.117.309715. 28360349 PMC5390330

[38] Wang Z, Zhao Y. 2018. Gut microbiota derived metabolites in cardiovascular health and disease. Protein Cell 9:416–431. doi:10.1007/s13238-018-0549-0. 29725935 PMC5960473

[39] Natarajan N, Hori D, Flavahan S, Steppan J, Flavahan NA, Berkowitz DE, Pluznick JL. 2016. Microbial short chain fatty acid metabolites lower blood pressure via endothelial G protein-coupled receptor 41. Physiol Genomics 48:826–834. doi:10.1152/physiolgenomics.00089.2016. 27664183 PMC6223570

[40] Bartolomaeus H, Balogh A, Yakoub M, Homann S, Markó L, Höges S, Tsvetkov D, Krannich A, Wundersitz S, Avery EG, Haase N, Kräker K, Hering L, Maase M, Kusche-Vihrog K, Grandoch M, Fielitz J, Kempa S, Gollasch M, Zhumadilov Z, Kozhakhmetov S, Kushugulova A, Eckardt K-U, Dechend R, Rump LC, Forslund SK, Müller DN, Stegbauer J, Wilck N. 2019. Short-chain fatty acid propionate protects from hypertensive cardiovascular damage. Circulation 139:1407–1421. doi:10.1161/CIRCULATIONAHA.118.036652. 30586752 PMC6416008

[41] Miao X, Liu C, Jiang Y, Wang Y, Kong D, Wu Z, Wang X, Tian R, Yu X, Zhu X, Gong W. 2021. BET protein inhibition evidently enhances sensitivity to PI3K/mTOR dual inhibition in intrahepatic cholangiocarcinoma. Cell Death Dis 12:1020. doi:10.1038/s41419-021-04305-3. 34716294 PMC8556340

[42] Carver BS, Chapinski C, Wongvipat J, Hieronymus H, Chen Y, Chandarlapaty S, Arora VK, Le C, Koutcher J, Scher H, Scardino PT, Rosen N, Sawyers CL. 2011. Reciprocal feedback regulation of PI3K and androgen receptor signaling in PTEN-deficient prostate cancer. Cancer Cell 19:575–586. doi:10.1016/j.ccr.2011.04.008. 21575859 PMC3142785

[43] Bhatt AP, Bhende PM, Sin SH, Roy D, Dittmer DP, Damania B. 2010. Dual inhibition of PI3K and mTOR inhibits autocrine and paracrine proliferative loops in PI3K/Akt/mTOR-addicted lymphomas. Blood 115:4455–4463. doi:10.1182/blood-2009-10-251082. 20299510 PMC2881502

[44] Chen J, Miao X, Liu C, Liu B, Wu X, Kong D, Sun Q, Gong W. 2020. BET protein inhibition prolongs cardiac transplant survival via enhanced myocardial autophagy. Transplantation 104:2317–2326. doi:10.1097/TP.0000000000003319. 32433238

